# Responses of maize hybrids to water stress conditions at different developmental stages: accumulation of reactive oxygen species, activity of enzymatic antioxidants and degradation in kernel quality traits

**DOI:** 10.7717/peerj.14983

**Published:** 2023-03-20

**Authors:** Muhammad Irfan Yousaf, Muhammad Waheed Riaz, Aamar Shehzad, Shakra Jamil, Rahil Shahzad, Shamsa Kanwal, Aamir Ghani, Farman Ali, Muhammad Abdullah, Muhammad Ashfaq, Quaid Hussain

**Affiliations:** 1 Cotton Research Station (CRS), Bahawalpur, Pakistan; 2 Maize and Millets Research Institute (MMRI), Yusafwala, Sahiwal, Pakistan; 3 State Key Laboratory of Subtropical Silviculture, Zhejiang A & F University, Hangzhou, China; 4 Zhejiang Provincial Key Laboratory of Resources Protection and Innovation of Traditional Chinese Medicine, Zhejiang A & F University, Hangzhou, China; 5 Maize Research Station, Ayub Agricultural Research Institute, Faisalabad, Pakistan; 6 Agricultural Biotechnology Research Institute, AARI, Faisalabad, Pakistan; 7 Queensland Alliance for Agriculture and Food Innovation, University of Queensland, Brisbane, Australia

**Keywords:** Chlorophyll, Osmotic stress, Photosynthesis, Proline contents, Protein contents, Transpirational rate

## Abstract

Sustainable maize production under changing climatic conditions, especially heat and water stress conditions is one of the key challenges that need to be addressed immediately. The current field study was designed to evaluate the impact of water stress on morpho-physiological, biochemical, reactive oxygen species, antioxidant activity and kernel quality traits at different plant growth stages in maize hybrids. Four indigenous *i.e*., YH-5427, YH-5482, YH-5395, JPL-1908, and one multinational maize hybrid *i.e*., NK-8441 (Syngenta Seeds) were used for the study. Four stress treatments (i) Control (ii) 3-week water stress at pre-flowering stage (iii) 3-week water stress at anthesis stage (iv) 3-week water stress at grain filling/post-anthesis stage. The presence of significant oxidative stress was revealed by the overproduction of reactive oxygen species (ROXs) *i.e*., H_2_O_2_ (1.9 to 5.8 µmole g^−1^ FW) and malondialdehyde (120.5 to 169.0 nmole g^−1^ FW) leading to severe negative impacts on kernel yield. Moreover, a severe reduction in photosynthetic ability (50.6%, from 34.0 to 16.8 µmole m^−2^ s^−1^), lower transpirational rate (31.3%, from 3.2 to 2.2 mmol m^−2^ s^−1^), alterations in plant anatomy, reduced pigments stability, and deterioration of kernel quality was attributed to water stress. Water stress affected all the three studied growth stages, the pre-flowering stage being the most vulnerable while the post-anthesis stage was the least affected stage to drought stress. Antioxidant activity was observed to increase under all stress conditions in all maize hybrids, however, the highest antioxidant activity was recorded at the anthesis stage and in maize hybrids YH-5427 *i.e*., T-SOD activity was increased by 61.3% from 37.5 U mg^−1^ pro to 60.5 U mg^−1^ pro while CAT activity was maximum under water stress conditions 8.3 U mg^−1^ pro as compared to 10.3 U mg^−1^ pro under control (19.3%). The overall performance of maize hybrid YH-5427 was much more promising than other hybrids, attributed to its higher photosynthetic activity, and better antioxidant defense mechanism. Therefore, this hybrid could be recommended for cultivation in drought-prone areas.

## Introduction

Field crops experience several types of stresses during their growth and development periods from sowing to harvesting under natural conditions. Among these biotic and abiotic stresses, drought or water stress is one of the more critical factors that determine crop productivity under unpredictably changing climatic conditions. About 80–95% of the fresh plant biomass is comprised of water, which plays a pivotal role in numerous physiological processes including photosynthesis, photorespiration, ATP synthesis, metabolism and several other aspects related to plant growth and development (Abbasi & Abbasi, 2010; Brodersen et al., 2019). Several researchers regard water stress as one of the greatest threats to the world’s food security in future and water stress served as the catalyst of great famines in the past (Okorie, Mphambukeli & Amusan, 2019). It was estimated that drought and floods accounted an approximately 80% of the total area affected by all natural disasters (Huang et al., 2022). The unpredictability of changing climatic conditions is increasing the frequency, intensity and duration of droughts in several parts of the world (Liu et al., 2020). Therefore, it is pivotal to study the responses of crops to changing climatic conditions to ensure food security.

Maize is one of the most important and diversified field crops due to its immense utilization in food, feed, fodder and biofuel industries. Moreover, it is also being widely used in dry and wet milling industries to provide flour, cooking oil, pharmaceuticals, glue, artificial sweetener, alcoholic beverages and starch. It is also used in the production of ethanol fuel (Ranum, Peña-Rosas & Garcia-Casal, 2014). In 2020–21, maize was cultivated on an area of 199.08 million hectares and 1,129.44 million metric tons production was obtained with an average of 5.67 metric tons per hectare (USDA, 2022). Although the kernel yield per hectare of maize in Pakistan (6.30 tons ha^−1^) is quite higher than the world’s yield (6.30 tons ha^−1^), it is still behind many countries including Turkey (11.45 metric tons ha^−1^), the United States (10.76 metric tons ha^−1^), Canada (9.63 metric tons ha^−1^), Egypt (8.00 metric tons ha^−1^), Argentina (7.94 metric tons ha^−1^), European Union (7.30 metric tons ha^−1^) (USDA, 2022). The major reasons for lower per hectare kernel yield in maize are high temperature, less availability of irrigation water, poor crop management, insufficient plant population, high input rates, insects and diseases infestations, less availability of hybrid seeds and selling of low-quality, substandard seed (Yousaf et al., 2021).

Water stress is one of the most detrimental environmental factors that severely affects maize growth and development. It induces several irreversible alterations at physiological, biochemical and molecular levels in maize. However, the severity of its effects depends upon the intensity, duration, cultivar and stage of plant development under stress. Several studies unveiled the drastic effects of water stress on physio-morphological, phonological, biochemical and molecular traits in maize (Sah et al., 2020; Pokhrel, 2021; Shemi et al., 2021; Seleiman et al., 2021; Yousaf et al., 2022). It is estimated that maize grain yield could be reduced from 10–27% due to a decrease in irrigation frequencies in maize (Leng, 2021). Similarly, in another study, the world’s maize yield and production are projected to decrease by 15–20% per year due to heat and drought stress conditions, as these two stresses are becoming a major threat to maize production (Hussain et al., 2019).

**Figure 1:**
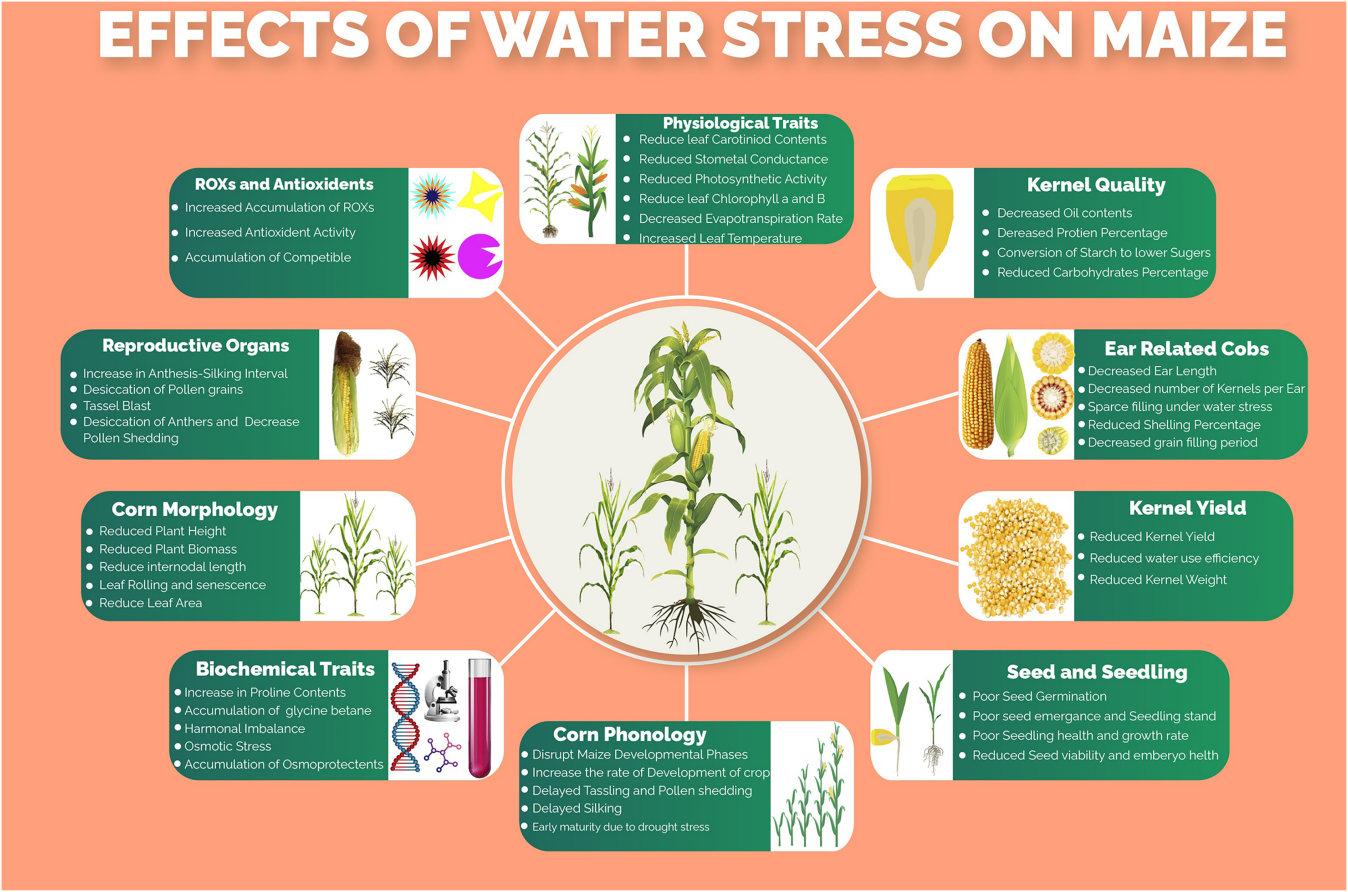
Effects of water stress on different morpho-physiological, phenological, biochemical and kernel quality related traits in maize.

Several morphological, phenological, physiological, biochemical, molecular and kernel quality-related traits and processes (Fig. 1) of maize are impaired under water stress conditions. Water stress was found to have drastic effects on plant height, ear height, number of kernels per ear, thousand kernels weight and ultimately the kernel yield per hectare (Sah et al., 2020). One of the major processes that are severely affected by water stress is photosynthesis. The key photosynthetic pigments including chlorophyll *a*, chlorophyll *b* and carotenoids markedly reduced under water stress conditions, resulting in decreased CO_2_ assimilation rate (Ashraf & Harris, 2013). Moreover, leaf transpiration, stomatal conductance and photosynthesis, which are tightly linked with maize productivity, are reduced under water stress thus hampering movements of nutrients in the plant, CO_2_/H_2_O movement through leaves and production of assimilates, respectively (Shafiq, Akram & Ashraf, 2019). Water use efficiency, which determines the grain production per unit of water consumed by the crop, is highly affected due to lower water ability and stomatal alteration under water stress conditions. The water stress, which resulted in osmotic stress, increased the production of reactive oxygen species (ROS) due to a decrease in molecular oxygen and generation of free radicals (Basu et al., 2016), which lead to lipid peroxidation, denaturation of cellular membranes, degradation of nucleic acids and proteins (Fathi & Tari, 2016). However, to scavenge the accumulation of these ROS, the plant starts to produce several enzymatic and non-enzymatic antioxidants including peroxidase (POX), superoxide dismutase (SOD), catalase (CAT), carotenoids, polyphenols, glutathione and ascorbate (Shafiq, Akram & Ashraf, 2019). Maize yield, oil and starch contents percentage were reported to be reduced under water stress conditions however the protein contents were getting increased (Correndo et al., 2021).

The present study was designed to evaluate the performance of maize hybrids under water stress at three key developmental stages. Moreover, the severity of maize developmental stages will be accessed based on several morpho-physiological and biochemical traits. The results obtained from this study will not only improve the understanding on impacts of drought stress on maize productivity and kernel quality but will also increase the farm yield and livelihood of farmers. The selected genotypes will be further processed for approval for general cultivation in drought-prone areas of the country.

## Materials and Methods

### Experimental material and site

This experimental study was conducted at the research area of Maize and Millets Research Institute, Yusafwala-Sahiwal (MMRI), Pakistan (longitude 73°12′39.3″E, latitude 30°40′57.1″E, altitude 173 m) under field conditions for two consecutive spring seasons (2019–2020 and 2020–2021). Seeds of three hybrids (YH-5482, YH-5427, and YH-5395) were obtained from the maize hybrid development group, Maize and Millets Research Institute, Yusafwala, while JPL-1908 was obtained from Jullundur Pvt. Ltd., Arifwala, and NK-8441 from Syngenta Seeds (Pakistan) respectively, during spring 2019–2020. About 1 kg seed per hybrid was stored at very low temperature and controlled humidity conditions in a cold store, installed at MMRI, Yusafwala to use in the next spring season (2020–2021). The field selected for the study had well-aerated, loamy sand textured soil having pH 7.8 and 16.51 g kg^−1^ soil organic matter.

### Water stress treatments

Maize hybrids were sown during mid-February under four conditions. (a) control (12 irrigations); (b) water stress at the pre-flowering stage (10 irrigations, two consecutive irrigations were held from 40 days after sowing); (c) water stress at the anthesis stage (10 irrigations, two consecutive irrigations were held from 60 days after sowing); (d) water-stress at anthesis stage (10 irrigations, two consecutive irrigations were held from 80 days after sowing). Hybrids were planted under randomized complete block design under split plot arrangement in triplicates. All other standard agronomic practices *i.e*., seed treatment, application of pre-emergence pesticide, insecticide for the control of shoot fly and maize borer. Recommended fertilizer application was given to all stress treatments at 92 kg nitrogen, 58 kg phosphorus, and 37 kg potash, respectively.

### Collection of samples for biochemical analysis

Three weeks after the stress was applied, three fully expanded, healthy cob leaves were taken from each replication. Following a distilled water wash, the leaves were frozen in liquid nitrogen (N_2_) and kept at −80 °C for the analysis of several biological components.

### Measurement of yield-related morpho-agronomical traits

Phenological traits *i.e*., days to 50% anthesis and days to 50% silking were measured as the days taken by the plant to complete their 50% anthesis and silking from date of sowing. Similarly, several morphological traits including plant and ear height, ear width, number of rows per ear, number of kernels per row, number of kernels per ear and thousand kernel weights were also measured during the experiment. These are important traits which were frequently used to study drought and heat stresses in maize by Yousaf et al. (2022). The one of the most important trait *i.e*., kernel yield per hectare is measured according to the formula given and used by Tandzi & Mutengwa (2022).

$${\rm Grain\; Yield\; }\left( {{\rm kg}/{\rm ha}} \right) = \displaystyle{{{\rm Fresh\; ear\; weight\; }\left( {{\rm kg}/{\rm plot}} \right) \times \left( {100 - {\rm MC}} \right) \times 0.8} \over {\left( {100 - 15} \right) \times Area{\rm \; }Harvested/plot}}{\rm \; } \times 10,\!000$$

where;

MC = moisture contents of kernels.

### Recording/measurement of physiological parameters and proline contents

Physiological parameters were analyzed from five, healthy and intact cob leaves per entry per treatment after impositions of drought stress treatments. The performance of maize hybrids under stress treatments was measured for three key physiological traits including net photosynthetic rate, stomatal conductance and transpiration rate using Infra-red gas analyzer (IRGA). The instrument used for the measurement was a handheld photosynthesis system (CI-320; CID Bio-Science Inc., Camas, WA, USA) as suggested and used by Yousaf et al. (2021). Moreover, the quantification of photosynthetic pigments *i.e*., chlorophyll *a*, *b* and carotenoid contents was done through spectroscopy using Shimadzu UV-1280 UV-VIS spectrophotometer (Peng & Liu, 1992; Lichtenthaler, 1987). The proline contents from maize leaves was quantified according to the procedure used by Efeoglu, Ekmekçi & Çiçek (2009).

### Accumulation of reactive oxidative species (MDA and H_2_O_2_)

The estimation or quantification of MDA through thiobarbituric (TBA) method given and used by Heath & Packer (1968) was used in this study. Similarly, H_2_O_2_ contents were determined according to the procedure developed by Alexieva et al. (2001). The reaction mixture consisting of leaf extract, 2.5 mM potassium-phosphate buffer (pH 7.0) and 0.5 M potassium iodide (KI). After keeping the mixture in the dark for 1 h, the amount of H_2_O_2_ was determined through a spectrophotometer at 415 nm by reference to a standard curve prepared with H_2_O_2_ solutions.

### Determination of enzymatic antioxidants (CAT and T-SOD)

The enzymatic activity of catalase (CAT) was measured by the method given by Chance & Maehly (1995) with a few modifications. After adding enzyme extract to start the reaction, its absorbance was measured every 20 s for 2 min at 240 nm. Each enzyme’s activity was expressed in terms of protein weight.

### Measurement of kernel quality traits

Kernel quality parameters, such as the percentages of kernel protein, oil, and starch, were assessed using near-infrared spectroscopy (NIR) with the use of an Inframatic 9200 from Partin Instruments in Sweden. For each replication, an average of three samples (750 g each) was utilised to calculate the kernel quality attributes’ contents. Percentages (%) were used to express the parameters.

### Statistical analysis

The recorded data for kernel yield and stress-associated traits were subjected to analysis of variance (ANOVA) and correlation coefficient analysis as suggested by Steel & Torrie (1997) through Statistix 8.1 (Analytical Software, Tallahassee, FL, USA) and XLSTAT (Addinsoft Inc, New York, NY, USA) statistical packages. The statistical differences between treatments were executed through Duncan’s multiple range test (DMRT). Biplot analysis was used to characterize maize hybrids concerning plant traits under different stress treatments by using XLSTAT 2020. Furthermore, OriginPro 2021 (OriginLab Corporation, Northampton, MA, USA) and Microsoft Excel 2019 were used for the graphical representation of data.

### Metrological data (spring 2019–2020 and 2020–2021)

**Figure 2:**
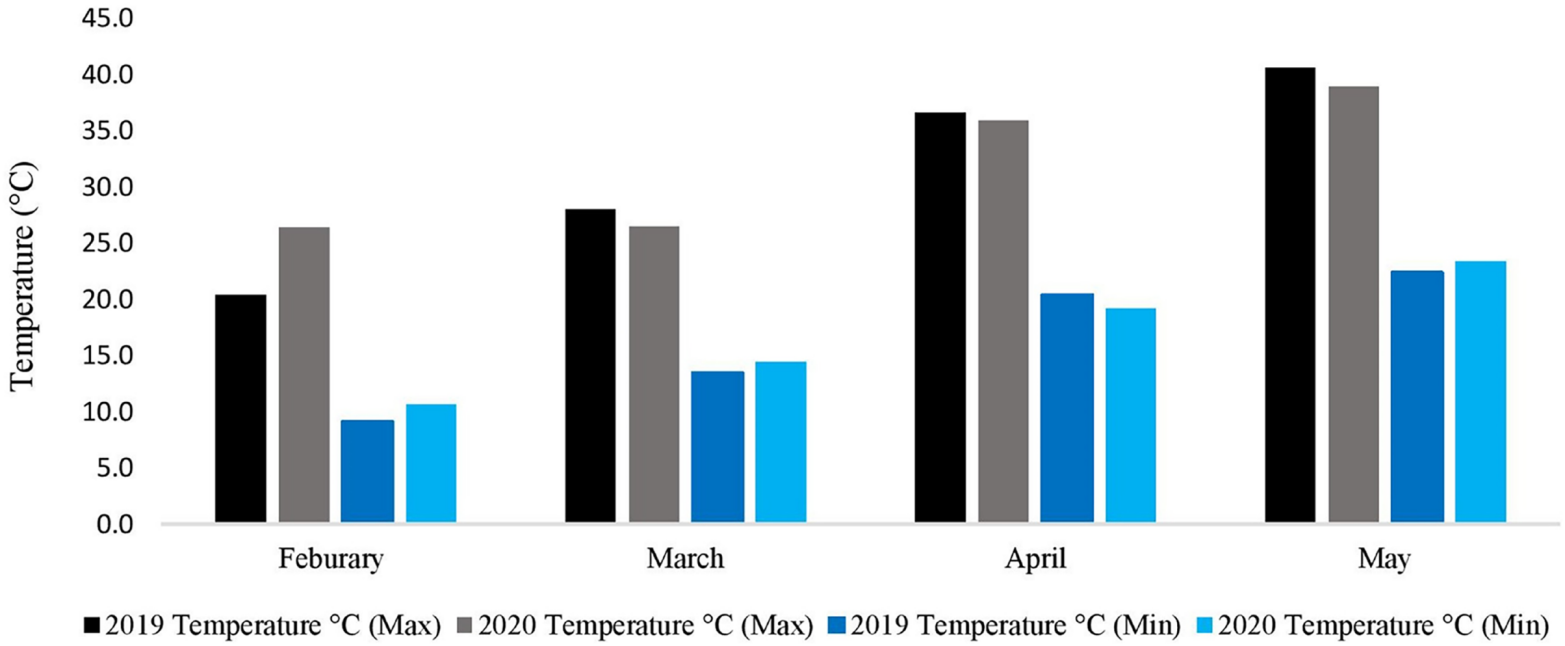
Metrological data of two spring growing seasons (2019–2020 & 2020–2021).

During the whole experimental study, data regarding metrological parameters *i.e*., minimum temperature/maximum temperature (°C), rainfall (mm), and relative humidity were recorded daily (Fig. 2). The data showed that the average daily maximum temperature was highest in May (40.6 °C and 38.9 °C) while the average daily minimum temperature was lowest in February (9.1 °C and 10.6 °C), during the spring growing season of 2019–2020 and 2020–2021, respectively. The rainfall data is not needed because the research area, where the experiment was conducted had the facility to cover the whole experimental area with a thick plastic sheet when needed. Indeed, the facility was used three times during the experiment to keep the rainfall away from the field.

## Results

### Kernel yield and related traits

The results derived from analysis of variance (ANOVA) revealed that a significant difference was present among the maize hybrids for maize kernel yield and its associated morpho-phenological traits *i.e*., days to 50% anthesis (DA), days to 50% silking (DS), plant height (PH), ear height (EH), ear width (EW), rows per ear (R/E), number of kernels per row (NK/R), number of kernels per ear (NK/E), thousand kernel weight (TKW) and kernel yield (KY) under different drought stress conditions (Table 1). Moreover, the interaction between drought stress treatments and maize hybrids was also significant for all the studied traits.

**Table 1:** Mean squares (MS) of plant traits in five maize hybrids under water stress conditions at different developmental stages.

Traits	Replication (R)	Years (Y)	Error (R × Y)	Treatments (T)	Error (Y × R × T)	Hybrids (H)	Interaction (T × H)	Error (R × Y × T × H)
Degree of freedom	2	1	2	3	12	4	12	48
Days to 50% anthesis	0.06	12.34	9.4	656.42**	0.12	26.35**	9.75**	0.25
Days to 50% silking	0.52	5.5	4.34	647.12**	0.15	26.84**	9.83**	0.31
Plant height	45.2	38.4	23.5	5,220.4**	19	934.5**	115.4**	6.8
Ear height	1.58	18.5	12.34	898.55**	1.35	945.6**	89.35**	1.5
Ear length	0.66	1.5	1.25	27.19**	0.05	4.98**	1.96**	0.09
Ear width	2.90	3.2*	1.53	248.19**	0.76	32.78**	6.12**	0.45
Rows per ear	0.52	0.324	0.18	13.77**	0.16	2.88**	1.25**	0.07
Kernels per row	2.5	1.34	0.9	81.3**	0.22	25.33**	11.67**	0.39
Kernels per ear	569.2	845.8	643.3	65,856.3**	71.5	3,807.3**	2,825.6**	101.3
Thousand kernels weight	209.1	174.7	95.2	21,883.2**	9.73	19,579.0**	1,471.7**	18
Net photosynthetic rate	0.58	0.35	0.21	262.86**	0.06	25.27**	7.92**	0.23
Protein %	0.24	0.357	0.27	5.59**	0.012	0.15**	0.32**	0.049
Oil %	0.031	0.473	0.349	0.491**	0.006	0.607**	0.085**	0SSS.007
Starch %	1.17	3.36	2.75	40.39**	0.41	68.89**	8.22**	0.55
Transpiration rate	0.007	0.0988	0.093	4.319**	0.004	0.258**	0.107**	0.003
Stomatal conductance	76.0	74.4	62.4	47,859.6**	31.9	8,251.3**	415.5**	14.6
Water use efficiency	0.22	0.284	0.17	38.91**	0.02	1.83**	1.35**	0.03
Chlorophyll *a*	0.233	0.074	0.066	1.232**	0.002	0.111**	0.046**	0.004
Chlorophyll *b*	0.003	0.05	0.041	0.273**	0.001	0.031**	0.017**	0.002
Carotenoids contents	0.0059	0.167	0.099	0.394**	0.001	0.068**	0.013**	0.003
Proline contents	0.35	0.96	0.7	872.72**	0.033	3.44**	2.48**	0.053
H_2_O_2_ contents	0.016	2.6	1.7	21.069**	0.009	0.005*	0.15	0.003
MDA contents	78.7	29.6	20.6	7,321.9	7.8	41.2	72.9	6.32
Total SOD (T-SOD)	9.39	23.4	16.7	2,967.34**	0.48	46.88**	16.45**	0.87
Catalysis (CAT)	0.16	3.2	2.8	29.49**	0.02	0.85**	0.26**	0.03
Kernel yield (kg ha^−1^)	5,492	6,186.5	5,322	27,291,287**	4,617	2,836,864**	444,836**	17,143

**Notes:**

Significant changes are highlighted by an asterisk (*).

* *P* ≤ 0.05.

** *P* ≤ 0.01.

The water stress at three critical plant development stages (pre-anthesis, anthesis, and post-anthesis) affected the overall development of the whole reproductive phase, which is, in fact, the most vulnerable stage to drought stress, causing a significant reduction in kernel yield and related traits (Table 2). Compared to control, water stress at the anthesis stage was proved more lethal than pre-anthesis and grain development stages as a significant reduction in most of the agro-morphological traits including days to 50% anthesis, days to 50% silking, ear width, rows per ear, number of kernels per row, number of kernels per ear, thousand kernel weight and kernel yield (Table 2). After the anthesis, water stress at pre-anthesis was the second most critical stage, severely affecting the plant and ear height of maize hybrids. However, ear length was harshly affected by the water stress at the grain development stage. Considering the performance of maize hybrids for morpho-phonological traits under control and water stress conditions at pre-anthesis, anthesis, and grain development stages, YH-5395 and YH-5427 outperform other maize hybrids *i.e*., YH-5482, NK-8441, and JPL-1908.

**Table 2:** Effects of water stress on morpho-physiological and yield-related parameters in maize hybrids at different plant developmental stages.

Hybrids	Treatment	DA	DS	PH	EH	EL	EW	R/E	NK/R	NK/E	GY	TKW
**YH-5482**	Control	74.1 ± 0.213	77.1 ± 0.223	178.3 ± 1.07	96.5 ± 0.736	18.9 ± 0.106	49.9 ± 0.410	17.5 ± 0.19	39.9 ± 0.272	699.4 ± 4.16	9,695 ± 57.2	288.8 ± 3.86
	Pre-anthesis	73.0 ± 0.225	76.0 ± 0.259	170.4 ± 1.95	90.7 ± 0.402	18.2 ± 0.109	45.7 ± 0.380	16.0 ± 0.26	38.2 ± 0.661	610.7 ± 6.25	6,489 ± 38.2	258.3 ± 0.52
	During-anthesis	72.3 ± 0.192	75.3 ± 0.168	168.4 ± 1.13	90.2 ± 0.512	18.0 ± 0.329	45.2 ± 0.567	15.8 ± 0.14	37.8 ± 0.269	596.5 ± 7.33	6,350 ± 77.2	255.5 ± 2.78
	Post-anthesis	73.7 ± 0.193	76.7 ± 0.171	175.3 ± 2.41	96.7 ± 1.289	17.0 ± 0.142	47.9 ± 0.594	16.7 ± 0.05	37.7 ± 0.211	626.0 ± 5.18	7,326 ± 21.2	251.4 ± 1.67
	Mean	73.3	76.3	173.1	93.5	18.0	47.2	16.5	38.4	633.2	7,465	263.5
**YH-5427**	Control	70.8 ± 0.413	73.6 ± 0.385	184.7 ± 0.63	94.2 ± 0.794	19.0 ± 0.052	47.0 ± 0.155	17.5 ± 0.40	39.7 ± 0.419	695.9 ± 8.00	9,671 ± 98.4	343.2 ± 1.41
	Pre-anthesis	73.2 ± 0.228	76.1 ± 0.262	157.6 ± 2.14	75.5 ± 0.559	17.9 ± 0.168	44.2 ± 0.143	16.8 ± 0.17	37.4 ± 0.208	628.4 ± 3.52	6,469 ± 67.4	315.5 ± 0.59
	During-anthesis	73.0 ± 0.330	76.0 ± 0.331	157.0 ± 1.15	76.2 ± 0.363	17.7 ± 0.312	43.7 ± 0.802	16.7 ± 0.10	37.4 ± 0.393	621.9 ± 14.92	6,664 ± 36.9	263.5 ± 2.15
	Post-Anthesis	70.7 ± 0.096	73.7 ± 0.106	171.7 ± 1.75	97.7 ± 0.339	17.7 ± 0.141	47.0 ± 0.585	16.7 ± 0.21	38.0 ± 0.380	633.3 ± 1.97	7,831 ± 19.9	309.6 ± 0.51
	Mean	71.9	74.9	167.8	85.9	18.1	45.5	16.9	38.1	644.9	7,659	308.0
**NK-8441**	Control	66.2 ± 0.237	69.2 ± 0.266	187.6 ± 1.97	86.2 ± 0.889	19.9 ± 0.256	47.7 ± 0.146	15.4 ± 0.09	39.0 ± 0.346	577.5 ± 11.41	9,510 ± 176.4	264.6 ± 1.75
	Pre-anthesis	69.2 ± 0.390	72.3 ± 0.301	163.9 ± 1.19	71.2 ± 0.465	17.8 ± 0.107	44.0 ± 0.481	14.5 ± 0.10	36.9 ± 0.244	516.3 ± 8.18	6,243 ± 24.3	220.4 ± 2.49
	During-anthesis	69.0 ± 0.196	72.0 ± 0.197	163.9 ± 2.19	71.2 ± 0.483	17.5 ± 0.091	43.7 ± 0.150	13.9 ± 0.10	36.3 ± 0.865	503.3 ± 3.71	6,113 ± 41.7	217.5 ± 3.76
	Post-anthesis	67.7 ± 0.335	70.3 ± 0.540	185.3 ± 2.04	91.3 ± 1.149	16.3 ± 0.009	43.5 ± 0.607	15.3 ± 0.21	36.7 ± 0.214	561.3 ± 3.23	7,113 ± 63.6	220 ± 4.73
	Mean	68.0	71.0	175.2	80.0	17.9	44.7	14.8	37.2	539.6	7,245	230.6
**JPL-1908**	Control	72.8 ± 0.422	76.0 ± 0.482	225.0 ± 3.07	90.0 ± 0.454	18.0 ± 0.226	46.8 ± 0.568	15.5 ± 0.40	38.3 ± 0.519	620.5 ± 4.19	8,478 ± 88.9	341.2 ± 4.67
	Pre-anthesis	68.3 ± 0.229	71.7 ± 0.222	179.6 ± 1.12	81.6 ± 0.397	17.4 ± 0.264	42.9 ± 0.383	14.8 ± 0.14	34.5 ± 0.430	510.1 ± 6.43	6,764 ± 43.2	310.7 ± 2.68
	During-anthesis	68.1 ± 0.197	71.1 ± 0.198	186.3 ± 2.22	81.9 ± 0.375	17.0 ± 0.122	42.8 ± 0.305	14.7 ± 0.16	34.1 ± 0.327	499.9 ± 9.00	6,194 ± 97.6	307.1 ± 2.92
	Post-anthesis	67.0 ± 0.332	70.0 ± 0.333	203.7 ± 1.92	87.3 ± 0.697	17.3 ± 0.199	45.5 ± 0.232	14.7 ± 0.05	30.7 ± 0.276	546.3 ± 4.31	6,959 ± 23.2	313.8 ± 0.73
	Mean	69.1	72.2	198.7	85.2	17.4	44.5	14.9	34.4	544.2	7,099	318.2
**YH-5395**	Control	74.5 ± 0.245	77.7 ± 0.246	201.8 ± 2.48	80.5 ± 0.563	20.6 ± 0.108	47.7 ± 0.586	16.8 ± 0.22	38.0 ± 0.480	662.1 ± 4.17	9,903 ± 160.7	367 ± 1.012
	Pre-anthesis	70.1 ± 0.229	73.1 ± 0.215	169.1 ± 0.57	71.1 ± 0.769	17.7 ± 0.159	44.3 ± −0.009	14.8 ± 0.17	35.2 ± 0.286	522.2 ± 1.92	7,180 ± 81.8	326.8 ± 3.47
	During-anthesis	69.8 ± 0.206	72.8 ± 0.179	174.5 ± 1.17	72.6 ± 0.306	17.5 ± 0.117	44.0 ± 0.321	14.6 ± 0.17	34.2 ± 0.293	497.6 ± 5.33	6,921 ± 86.5	321.6 ± 3.67
	Post-anthesis	67.7 ± 0.194	70.7 ± 0.462	184.3 ± 2.20	80.0 ± 0.281	17.0 ± 0.173	45.6 ± 0.374	14.7 ± 0.17	33.0 ± 0.126	543.7 ± 3.08	7,812 ± 55.14	326.9 ± 2.36
	Mean	70.5	73.6	182.4	76.1	18.2	45.4	15.2	35.1	556.4	7,954	335.6

**Note:**

The values are obtained by pooling the mean data of three replicates of two spring growing seasons (2019–2020 & 2020–2021). ±SE (Standard Error); DA, Days to 50% anthesis; DS, Days to 50% silking; PH, Plant height (cm); EH, Ear height (cm); EL, Ear length (cm); EW, Ear width (mm); R/E, Number of kernel rows per ear; NK/R, Number of kernels per row; NK/E, Number of kernels per ear; GY, Grain yield per hectare (kg ha^−1^); TKW, Thousand Kernel Weight (g).

**Figure 3:**
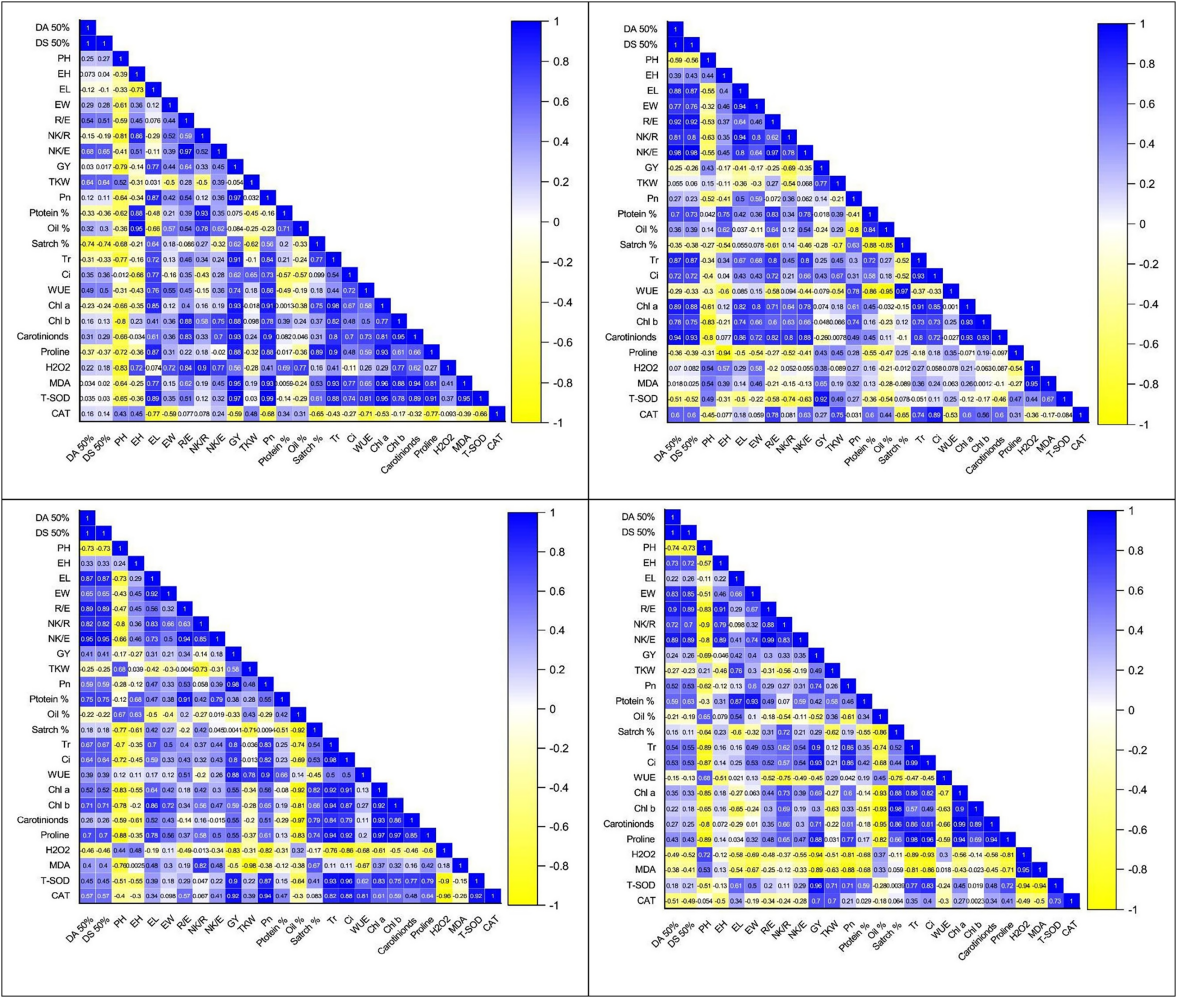
Correlation coefficient graphs of morpho-physiological, biochemical and stress-related parameters in five maize hybrids under water stress under (A) control (B) pre-anthesis (C) during-anthesis (D) post-anthesis.

The correlation coefficient analysis revealed a significantly positive correlation of kernel yield with EL (0.77**), R/E (0.64**), NK/E (0.45*), EW (0.44*), and NK/R (0.33*) while significantly negative with PH (−0.79**), under control (Figs. 3A–3D). However, this correlation between morpho-phonological traits got changed in water stress conditions. There was a negative but non-significant correlation of KY with DT (−0.25^ns^) and DS (−0.26^ns^) under water stress at the anthesis stage, which was positive yet non-significant (0.030^ns^, 0.017^ns^) under control, respectively (Figs. 3A–3D). The correlation between the same traits under water stress conditions at the grain development stage was also positive (0.24^ns^, 0.26^ns^) but significant only for the anthesis stage (0.41*, 0.41*), respectively (Figs. 3A–3D). Similarly, the correlation of GY with EW, R/E, and NK/E were positive under control (0.44*, 0.64**, 0.45*), anthesis (0.21^ns^, 0.34^ns^, 0.18^ns^), and grain development stage (0.40*, 0.30^ns^, 0.35^ns^), while negative under pre-anthesis water stress condition (−0.17^ns^, −0.25^ns^, −0.35^ns^). The correlation of KY with PH was significantly positive under only the pre-anthesis water stress condition (0.43*) while negative under the control (−0.79**), anthesis (−0.17^ns^), and grain development stages (−0.69*), respectively (Figs. 3A–3D).

### Physiological traits and photosynthetic pigments

The results obtained from ANOVA unveiled the presence of highly significant differences (*P* ≤ 0.05) among maize hybrids, stress treatments, and their interactions for physiological traits and photosynthetic pigments *i.e*., net photosynthetic rate (Pn), stomatal conductance (Ci), transpirational rate (Tr), chlorophyll *a* and *b* (Chl *a*, Chl *b*) and carotenoid contents (carotenoid) (Table 1).

**Figure 4:**
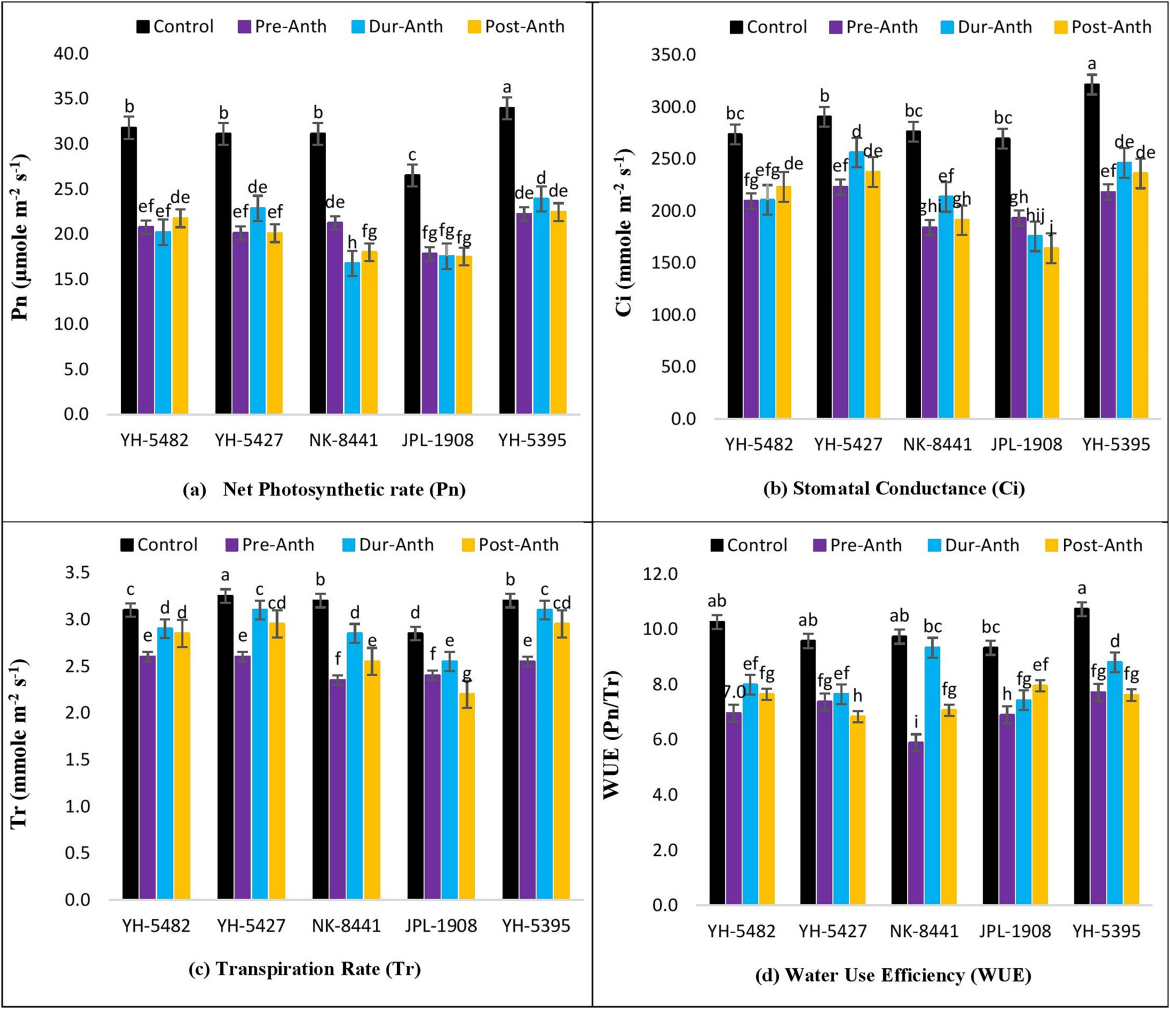
Effect of water stress on (A) net photosynthetic rate (B) stomatal conductance (C) transpirational rate and (D) water use efficiency in five maize hybrids. Capped bars above means represent ±SE of three replicates and English letters represent their statistical significance.

**Figure 5:**
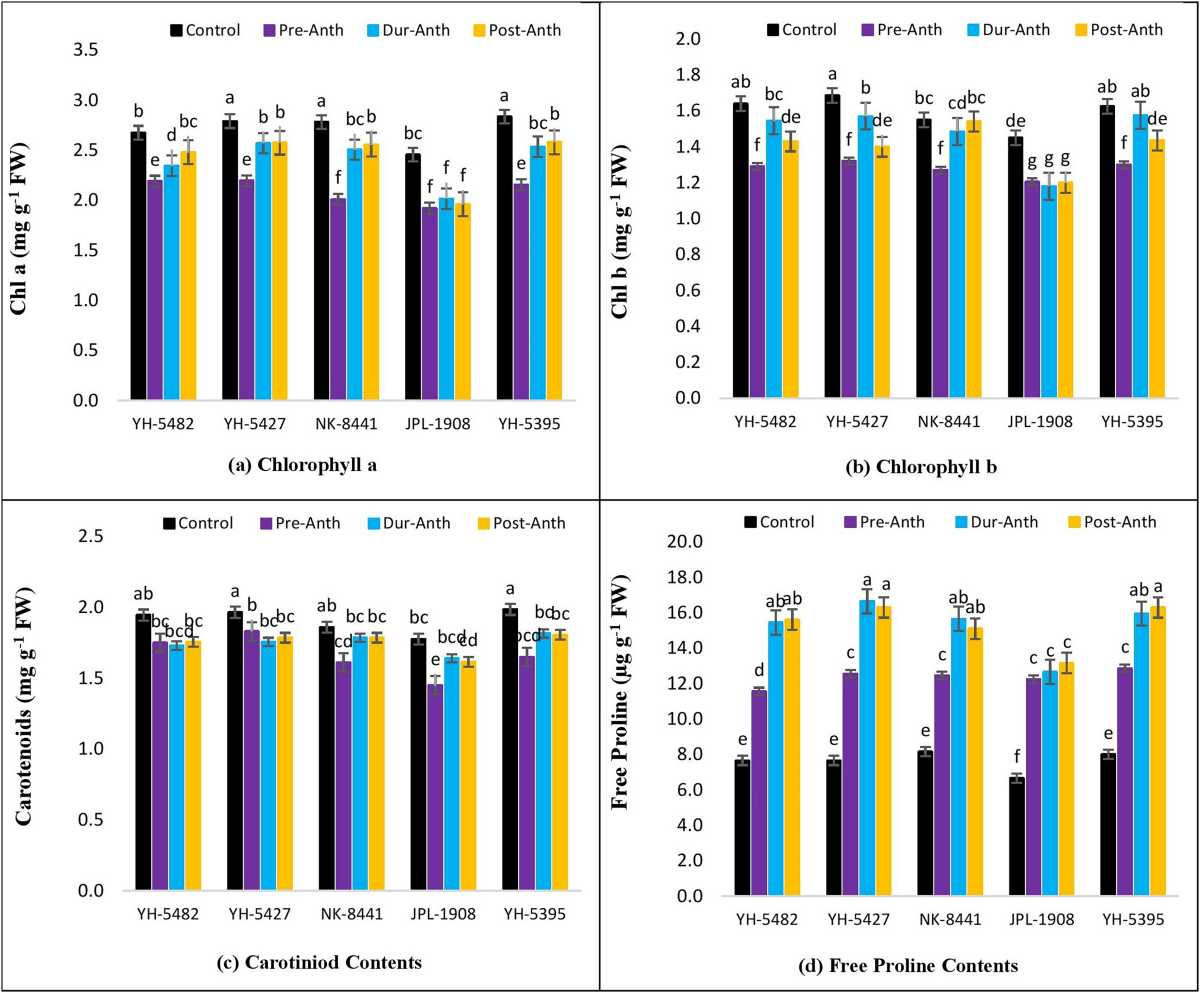
Effect of water stress on (A) chlorophyll *a* (B) chlorophyll *b* (C) carotenoid contents and (D) free proline contents in five maize hybrids. Capped bars above means represent ±SE of three replicates and English letters represent their statistical significance.

The impact of water stress on different plant physiological parameters at different growth stages was quite lethal, hindering the key physiological processes (Figs. 4A –4D and 5A–5D). The results revealed a significant reduction in major physiological traits and photosynthetic pigments *i.e*., net photosynthetic rate, stomatal conductance, transpirational rate, water use efficiency, chlorophyll *a* and *b*, and carotenoid contents under water-stress conditions during pre-anthesis, anthesis and grain development stages. Compared to control, water stress during the pre-anthesis stage was the most crucial period that resulted in the severe reduction of all the physiological traits and photosynthetic pigments. However, the impact of water stress during the anthesis phase was also at par with pre-anthesis water stress for physiological parameters. In maize hybrids, the net photosynthetic rate (Pn) was the most affected physiological trait under water stress conditions. Similarly, the negative effects of water stress on photosynthetic pigments in maize hybrids were significantly high except for carotenoid contents, which were less affected by water stress across the developmental stages. Leaf chlorophyll contents *i.e*., chlorophyll *a* and *b* (Chl *a* and Chl *b*) were significantly reduced under water stress conditions at the pre-anthesis stage (Figs. 5A–5D).

However, the impact of water stress during anthesis and grain development stages was statistically non-significant.

Considering the performance of maize hybrids under water stress conditions, two locally developed maize hybrids *i.e*., YH-5395 and YH-5427 appeared to be more drought tolerant than the other contending hybrids due to their relatively stable performance under stress conditions compared to other hybrids. However, their performance regarding the stability of photosynthetic pigments under water stress conditions was at par with a famous multinational maize hybrid NK-8441 of Syngenta Seeds.

Correlation analysis showed a varying degree of association between kernel yield and physiological traits and photosynthetic pigments under water stress conditions at different growth stages (Figs. 4A –4D and 5A–5D). The data revealed that kernel yield had a significantly positive correlation with net photosynthetic rate, stomatal conductance, transpirational rate, and chlorophyll-*a* under all water stress conditions. However, the correlation between these traits was a relatively quiet week at the pre-anthesis stage. Moreover, water use efficiency had a significantly positive correlation with kernel yield under control (0.74**) and anthesis water stress stage (0.88**), while negative under water stress at pre-anthesis (−0.079^ns^) and grain development stage (−0.45*), respectively (Figs. 3A –3D). Similarly, chlorophyll *b* was positively correlated with kernel yield under control (0.88**) and water stress treatment at anthesis (0.59**) and grain development (0.30^ns^) stages while negatively correlated at pre-anthesis water stress conditions (−0.048^ns^) (Figs. 3A –3D).

### Free proline contents

The analysis of variance showed the presence of significant genetic variation among maize hybrids for free proline contents under water stress conditions (Table 1). The results revealed that free proline contents were increased under water stress conditions in maize hybrids (Fig. 5D). Compared to control, the minimum effects of water stress were observed in the pre-anthesis stage, while the maximum increase of free proline contents was recorded in the grain development stage, indicating the susceptibility of the grain development stage to water stress. The differences between two water stress treatments *i.e*., anthesis and grain development were statistically non-significant. The correlation analysis also unveiled a strong positive correlation of free proline contents with kernel yield under control (0.88**) and water stress at different plant growth stages *i.e*., pre-anthesis (0.43*), anthesis (0.55*), and grain development (0.88**), respectively (Figs. 3A –3D).

### Accumulation of reactive oxygen species (ROXs)

**Figure 6:**
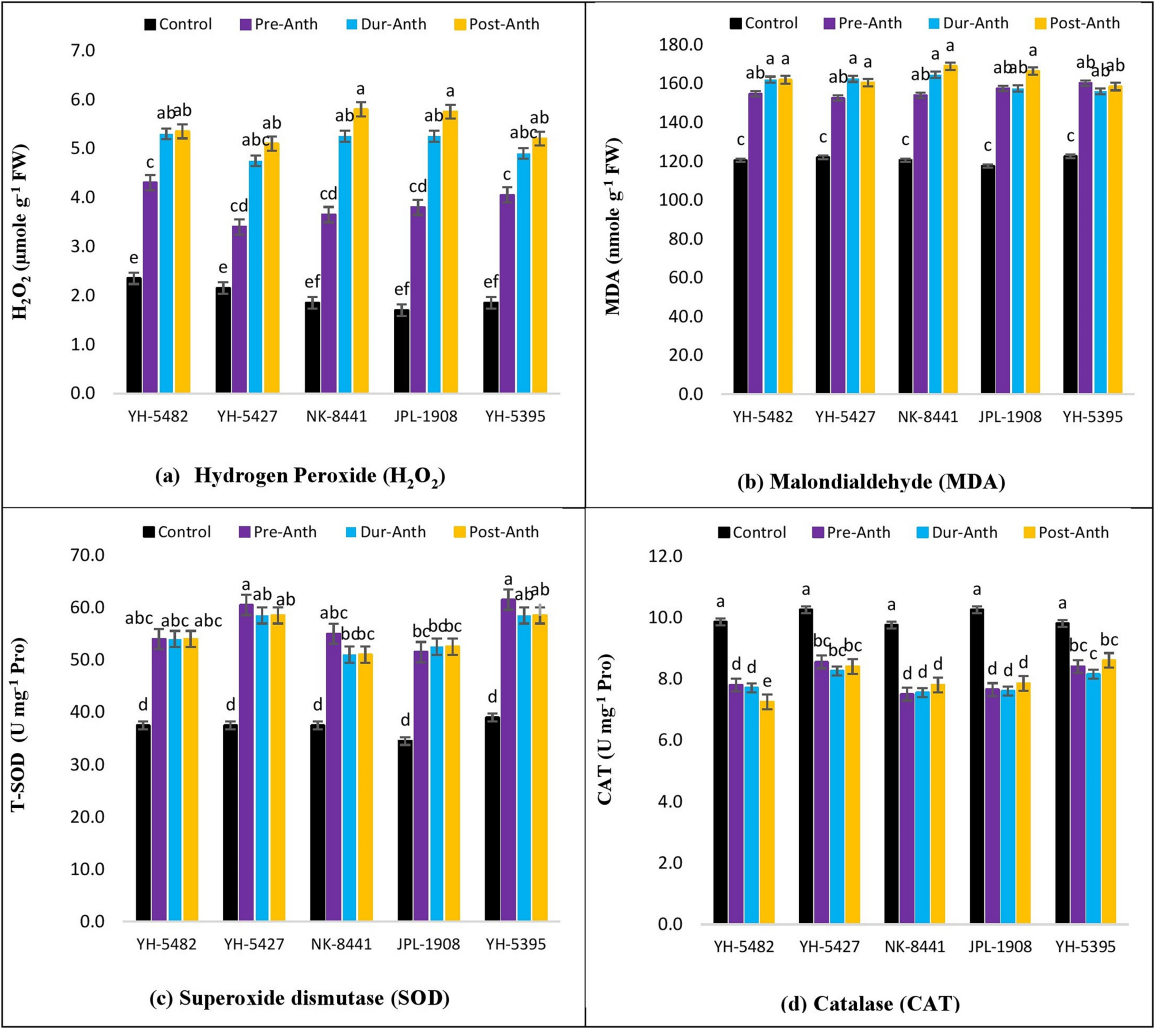
Effect of water stress on (A) H_2_O_2_ (B) MDA (C) SOD and (D) CAT in five maize hybrids. Capped bars above means represent ±SE of three replicates and English letters represent their statistical significance.

An increase in the accumulation of reactive oxygen species (ROXs) is one of the key responses of maize crops under osmotic/oxidative stress. Analysis of variance revealed significant differences among maize hybrids, stress treatments and their interactions for accumulation of ROS *i.e*., hydrogen peroxide (H_2_O_2_) and malondialdehyde (MDA) (Table 1). However, the variations were non-significant concerning years and their interactions with other sources of variations. Further results indicated a significant increase in H_2_O_2_ and MDA accumulation under water stress conditions (Figs. 6A –6D). Considering the accumulation of ROS under water stress conditions at different maize developmental stages, the highest accumulation of H_2_O_2_ and MDA was observed at the post-anthesis stage (post-anth) followed by the anthesis stage (during anthesis). The highest accumulation of H_2_O_2_ was recorded in NK-8441 and JPL-1908 (5.8 µmole g^−1^ FW) followed by YH-5482 (5.4 µmole g^−1^ FW) at post-anthesis stage while the lowest increase in ROS activity was observed in YH-5427 (5.1 µmole g^−1^ FW). Similarly, the highest accumulation of MDA was recorded in NK-8441 (169.0 nmol g^−1^ FW) followed (166.5 nmol g^−1^ FW) at the post-anthesis stage while the lowest accumulation in YH-5427 (152.5 nmol g^−1^ FW) at the pre-anthesis stage, respectively (Figs. 6A –6D). The correlation analysis unveiled a strong negative correlation of kernel yield with H_2_O_2_ and MDA under water stress at anthesis (r = −0.83**, r = −0.5*) and post-anthesis (r = −0.94**, r = −0.89 **), respectively (Figs. 3A–3D).

### Accumulation of enzymatic antioxidants

With an increase in the production of reactive oxygen species (ROXs), plant triggers several mechanisms to protect cell components. Among these mechanisms, the production of antioxidants is one the most important mechanisms to scavenge and detoxify ROS species. In the current study, significant differences among maize hybrids, stress treatments and their interactions for the accumulation of antioxidants *i.e*., superoxide dismutase (SOD) and catalase (CAT) were found through analysis of variance (Table 1). However, the variations were non-significant concerning years and their interactions with other sources of variations.

Further results indicated a significant increase in SOD and CAT accumulation under water stress conditions (Figs. 6A –6D). The highest accumulation of SOD was observed during the water stress at the pre-anthesis stage (pre-anth) while for CAT, the highest activity was recorded during water stress at pre-anthesis and post-anthesis, respectively (Figs. 6A –6D). Concerning the performance of maize hybrids regarding the activity of antioxidants, the highest SOD and CAT accumulation was observed in YH-5395 (61.5 U mg^−1^ pro, 60.5 U mg^−1^ pro) and YH-5427 (8.6 U mg^−1^ pro, 8.6 U mg^−1^ pro). The correlation analysis unveiled a strong positive correlation of kernel yield with SOD and CAT under water stress at pre-anthesis (r = 0.92**, r = 0.27^ns^), during anthesis (r = 0.90**, r = 0.92**) and post-anthesis (r = 0.96**, r = 0.70**) stages, respectively (Figs. 3A–3D).

### Kernel quality traits

**Figure 7:**
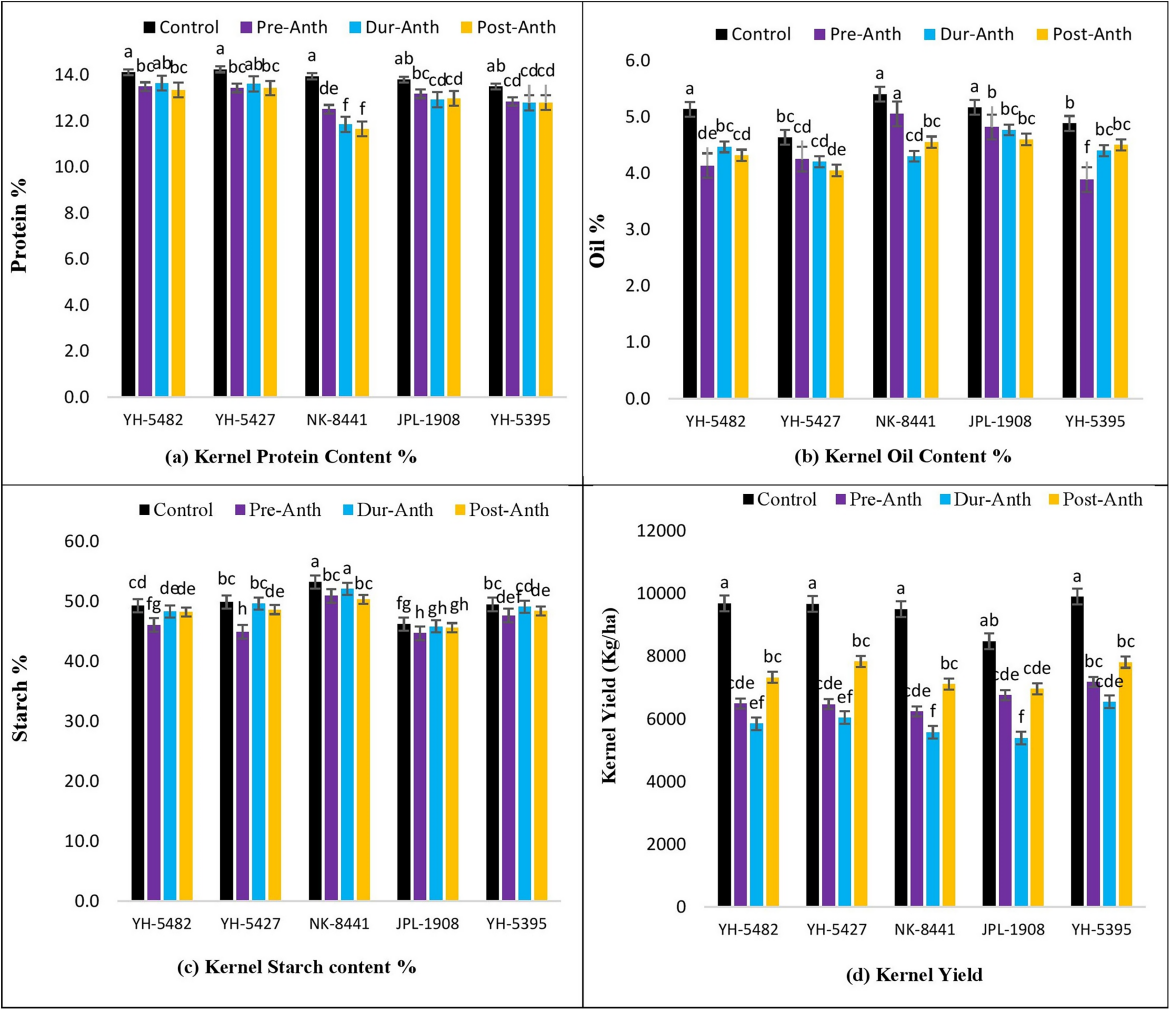
Effect of water stress on (A) kernel protein contents % (B) kernel oil contents %, (C) kernel starch contents % (D) kernel yield in five maize hybrids. Capped bars above means represent ±SE of three replicates and English letters represent their statistical significance.

Analysis of variance revealed significant variations among maize genotypes for kernel quality traits across the treatments (Table 1). The results unveiled the significantly damaging effects of water stress (WS) on kernel quality traits at different maize development stages (Figs. 7A–7D). A significant reduction in kernel protein content percentage was observed in maize hybrids, especially NK-8441, where protein % decreased from 14.0% (Control) to 11.7% (WS at post-anthesis) (Figs. 7A–7D).

Among the crop development stages, water stress at the post-anthesis stage proved lethal for kernel protein content percentage. Similarly, kernel oil contents percentage was also highly affected by the water stress conditions at different stages (Figs. 7A–7D). The maximum oil % was observed in NK-8441 (5.4% at the control) while YH-5395 gave the minimum oil % (3.9%). The maximum decline in oil % was reported in YH-5482 and YH-5395 (25%) (Figs. 7A–7D). The impact of water stress at different plant stages for oil % was random. Water stress also affects the kernel starch content percentage which is another very important maize kernel quality parameter. The highest starch % was observed in exotic, multinational hybrid NK-8441 (53.3%) at control while the lowest in the JPL-1908 (44.7%) during water stress at the pre-anthesis stage (Figs. 7A–7D). The maximum reduction in starch percentage (10.8%) was observed in maize hybrid YH-5427, from 49.9% in control to 45.0% at pre-anthesis water stress (Figs. 7A–7D). The highest impact of water stress was recorded in the pre-anthesis stage, where reduction in starch percentage ranged from 3% to 10.8%.

## Discussion

Sustainable crop production under unpredictably changing climatic conditions is the most crucial challenge for crop scientists to feed immensely growing human populations. These conditions are further intensified by the deforestation, urbanization, salinization and desertification of available cropland. Among the climatic factors, heat and water stresses are the key factors responsible for lower crop yields and unsustainability in their production. Water or drought stress is one of the major contributors to reduced crop productivity which ultimately affects millions of people. Nearly 55 million people across the globe are affected by the droughts, imposing a serious threat to livestock and crops (WHO, 2022). In the current study, a significantly negative impact was observed on several agro-morphological traits including plant height, ear height, ear length, ear width, number of rows per ear, number of kernels per row, number of kernels per ear, thousand kernel weights and ultimately kernel yield per hectare. It was observed that the negative effects of water stress were much severer in pre-anthesis and during anthesis stages while the post-anthesis stage was less affected by the water stress conditions. The major reason is that pre-anthesis and during-anthesis stages were the stages of maize vegetative growth which are supposed to be the stages with the highest biomass accumulation (Bhattacharya, 2019). As water stress reduced the total biomass of the plant by decreasing internodal length and plant height, which resulted in an imbalance in the source-sink ratio, resulting in poor biomass portioning and weaker sink (Ge et al., 2012). This will increase the understanding on biomass portioning and stem reserves mobilization under drought stress conditions at late vegetative and early reproductive stages. That is why, serval kernel-related traits including ear length, ear width, number of rows per ear, number of kernels per row, number of kernels per ear, thousand kernel weights and kernel yield per hectare were badly influenced by the water stress at pre-anthesis and during-anthesis stages (Sah et al., 2020). Several previous studies also elucidated the negative effects of water stress on maize genotypes based on morpho-phonological traits (Sah et al., 2020; Dordas et al., 2018; Moharramnejad et al., 2019). However, the extent of damage due to water stress varies greatly with the intensity of stress, stage of crop development and cultivars (Fahad et al., 2017). Among maize hybrids, YH-5482 and YH-5395 out-performed other hybrids under water stress conditions while JPL-1908 and NK-8441 were proved to be fairly drought susceptible hybrids based on their agro-morphological traits. These hybrids (YH-5482 and YH-5395) were also proved as heat tolerant hybrids in the previous studies (Rahman et al., 2022). Therefore, these hybrids could be distributed in large quantities to the farming communities in drought prone areas to improve their livelihood.

In the current study, it has also been revealed that water stress has deleterious effects on key physiological traits including net photosynthetic rate, stomatal conductance, transpirational rate and water use efficiency at different maize developmental stages. Significant reduction in physiological traits was one of the major reasons for poor crop productivity under water stress conditions. A significant reduction in the net photosynthetic rate was observed in all three water stress treatments as compared to control in maize hybrids. The primary cause for reduced photosynthesis was the closure of stomata which decreased the amount of CO_2_ entering stomata (Xu et al., 2016). Stomatal conductance is the key trait that controls the photosynthesis, photorespiration and water use efficiency in maize crops. In the present study, water stress significantly reduced the stomatal conductance due to alterations in stomatal size, aperture and densities (Bertolino, Caine & Gray, 2019). Transpirational rate and water use efficiency of maize hybrids were also greatly reduced under water stress conditions mainly due to the alteration s in stomatal properties (Ullah et al., 2019). Furthermore, the impact of water stress on transpirational rate and water use efficiency was much severer at the pre-anthesis stage compared to the during-anthesis and post-anthesis stages. Maize hybrids responded quite differentially regarding the performance of physiological traits under water stress conditions. The performance of two maize hybrids *i.e*., YH-5395 and YH-5427 was quite promising regarding their efficiency to cope with water stress conditions and sustain their physiological performance with minimum damage. These hybrids also proved their performance under heat stress conditions in previous studies (Ghani et al., 2020).

The harmful effects of water stress on photosynthetic pigments *i.e*., chlorophyll *a*, chlorophyll *b* and carotenoid contents were also evident in the current study. Significant differences were observed among maize hybrids and growth stages for chlorophyll *a*, chlorophyll *b* and carotenoid contents under water stress conditions. The impact of water stress was more deleterious in the pre-anthesis stage compared to during-anthesis and post-anthesis stages. Overall the degradation of chlorophyll *a* and *b* was maximum in JPL-1908 while YH-5427 and YH-5395 sufficiently tolerate the negative effects of water stress on chlorophyll *a* and *b*. That was the major reason for higher photosynthetic activity and kernel yield in these two hybrids (Sherin, Aswathi & Puthur, 2022). In comparison to chlorophyll *a* and *b*, the negative effects of water stress on carotenoid contents were lower. However, water stress at the pre-anthesis stage was again lethal on maize hybrids. Proline, an amino acid, is an excellent marker/indicator of water stress in plants. Accumulation of proline increases with the increase in water stress (Dash et al., 2022). In the current study, a significant increase in proline accumulation was observed under all three water stress conditions. However, the maximum accumulation was recorded during anthesis and post-anthesis water stress conditions in maize hybrids. The highest accumulation of proline was recorded in YH-5427 and YH-5395 while the lowest was in JPL-1908. The higher accumulation of proline in YH-5427 and YH-5395 was one of the reasons behind osmotic stress tolerance and supply of ATPs under stress (Furlan et al., 2020).

The accumulation of reactive oxygen species (ROS) under water stress is the key factor of osmotic stress experienced by the maize hybrids. Although the accumulation of ROS is kept under tight control by various plant defense mechanisms, however, the increase in ROS levels under water stress conditions serves as an alarm signal that triggers several acclimatory responses through signal transduction pathways involving hydrogen peroxide as a secondary messenger (Cruz de Carvalho, 2008). In the current study, the accumulation of hydrogen peroxide (H_2_O_2_) and malondialdehyde (MDA) was increased to many folds under water stress conditions regardless of the stage of stress applied. The highest accumulation of H_2_O_2_ and MDA under water stress was observed at the post-anthesis development stage while the lowest activity was recorded at the pre-anthesis stage. Comparing the performance of maize hybrids based on ROS accumulation, YH-5427 and YH-5395 showed maximum stability to water stress conditions compared to other hybrids. The lowest H_2_O_2_ and MDA activity in these hybrids depict the stronger stress response mechanisms to combat fetal effects caused by oxidative stress (Cruz de Carvalho, 2008). Similar results were also reported by Hussain et al. (2019) and Yousaf et al. (2022) who explained the increased activity of H_2_O_2_ and MDA under heat and water stress conditions. On the other side, the hybrids, in which the ROS activity was found at maximum *i.e*., NK-8441 and JPL-1908 also appeared to be the least productive under stress conditions.

Water stress tolerance is a cellular homeostatic achieved through a delicate balance between ROS production and their elimination by the production of various types of antioxidants. When this delicate balance is disturbed because of any stress, the accumulation of ROS increases. To main the balance and cope with the drastic effects of osmotic stress caused by the increased ROS accumulation, the plant starts to increase the activity of several enzymatic and non-enzymatic antioxidants. In the present study, the response of two enzymatic antioxidants *i.e*., superoxide dismutase (SOD) and catalase (CAT) was examined under water stress at different developmental stages of maize hybrids. A significant increase in antioxidant activity was observed in all hybrids and in all water stress conditions compared to control. Maximum SOD and CAT activity was recorded at the post-anthesis water stress stage while minimum accumulation was observed at water stress during-anthesis the stage. Although, the differences in SOD and CAT accumulation under stress treatments during all three maize development stages were statistically non-significant, however strong significant variations were present between control and water stress treatments. Comparable results were also reported by Hussain et al. (2019) and Yousaf et al. (2022) who reported the increase in SOD and CAT activity due to the result of osmotic stress caused by heat and water stress conditions in maize. Comparing the performance of maize hybrids based on the increased antioxidant activity under water stress, two maize hybrids, YH-5427 and YH-5395 outperformed other hybrids by maintaining higher antioxidant activity. Higher antioxidant activity in these hybrids was also reported under heat stress conditions (Yousaf et al., 2022).

As maize is primarily used in the poultry industry for feed, the kernel quality must be considered while evaluating the genotypes. In the current study, the response of maize hybrids to water stress conditions was examined on three key kernel quality traits *i.e*., kernel protein contents percentage (protein %), kernel oi contents percentage (oil %) and kernel starch contents percentage (starch %). A significant decrease in protein, oil and starch contents percentage was recorded under water stress conditions at all three plant growth stages concerning control. The highest impact of water stress on protein % was observed in maize hybrid NK-8441 while minimum effects were recorded in YH-5427 and YH-5482. Similar results were also reported by Barutçular et al. (2016) and Sehgal et al. (2018) who reported the decrease in protein contents of maize kernels under water stress conditions. However, several other studies suggested the increase in protein contents after the longer exposure of maize genotypes to water stress conditions (Rakszegi et al., 2019; Nagy-Réder et al., 2021). Similarly, a considerable reduction in oil and the starch percentage was observed in all maize hybrids under water stress. However, the highest reduction in oil and the starch percentage was observed in YH-5427 and JPL-1908, respectively. On the other hand, JPL-1908 and NK-8441 were the hybrids which showed maximum oil and starch percentage under water stress conditions, respectively. Concerning water stress at growth developmental stages, stress at the pre-anthesis stage was proved to be more lethal than water stress during and post-anthesis stage.

## Conclusions

The current field study inferences the quantitative impact of water stress on morpho-physiological, biochemical, reactive oxygen species, antioxidant activity and kernel quality traits at different plant growth stages in maize hybrids. Water stress was observed to have strong negative effects on photosynthetic ability, transpirational rate, water use efficiency, stomatal conductance, reduced photosynthetic pigments stability, and deterioration of kernel quality. Moreover, water stress significantly increased the production of reactive oxygen species (ROS) *i.e*., H_2_O_2_ and MDA. In response to the increased accumulation of ROS, the activity of two enzymatic antioxidants, SOD and CAT were increased significantly to scavenge and detoxify the negative effects of oxidative stress. The overall performance of maize hybrid YH-5427 was much more promising than other hybrids, attributed to its higher photosynthetic activity, better antioxidant and osmolyte-based defense mechanism, stability of leaf pigments, and minimum reduction in kernel yield-related agronomic traits. Therefore, this hybrid could be recommended for cultivation in drought-prone areas.

## Supplemental Information

10.7717/peerj.14983/supp-1 Supplemental Information 1 Raw data.

## Additional Information and Declarations

### Data Availability

The following information was supplied regarding data availability: The raw data is available in the Supplemental Files.
